# An Epigastric Heteropagus Twin with Ruptured Giant Omphalocele

**Published:** 2014-04-01

**Authors:** Sajid Hameed Dar, Naeem Liaqat, Javaid Iqbal, Tariq Latif, Asif Iqbal

**Affiliations:** Paediatric Surgery Department, Services Hospital, Lahore, Pakistan

**Keywords:** Twin, Heteropagus, Omphalocoele, Epigastric

## Abstract

We present a case of heteropagus twins attached to the epigastric region. The neonate also had ruptured giant omphalocoele with most of gut and liver lying outside the abdominal cavity. Patient had uneventful surgery for separation of twins and repair of ruptured omphalocoele.

## INTRODUCTION

Conjoint twinning is a rare entity with occurrence rate of 1 in 50,000 to 1 in 100,000 live births.[1] When one in the conjoint twins is having major congenital anomalies and is attached to a normal looking fetus, the set is said to be asymmetrical or Heteropagus.[2] We present a case of epigastric heteropagus twins with ruptured giant omphalocele.

## CASE REPORT

A full-term male baby born to a 30 years old primigravida through spontaneous vaginal delivery referred to us with anterior abdominal wall defect and four extra limbs with small trunk attached to the epigastric area. The parasite twin was attached to the right side of xiphoid sternum in the epigastrium and had well-formed mermaid like fused lower limbs. It also had hypoplastic empty scrotum and rudimentary upper limbs. There was a ruptured giant omphalocoele, 10 x 10 cm, just below the attachment of parasite. Most of the gut and liver were eviscerated (Fig. 1). The eviscerated viscera were reducing and wound was dressed. His preoperative work up including ultrasound abdomen and echocardiography were unremarkable. After resuscitation, patient was operated. During abdominal exploration, an isolated 1.5 feet long part of intestine was found attached to the superior surface of the liver which was excised in toto. The parasitic twin was separated with borrowing of its skin for the closure of the wound. Only skin closure was possible after reducing eviscerated viscera. His post-operative recovery remained uneventful. The patient is doing well at follow-up (Fig. 2).

**Figure:**
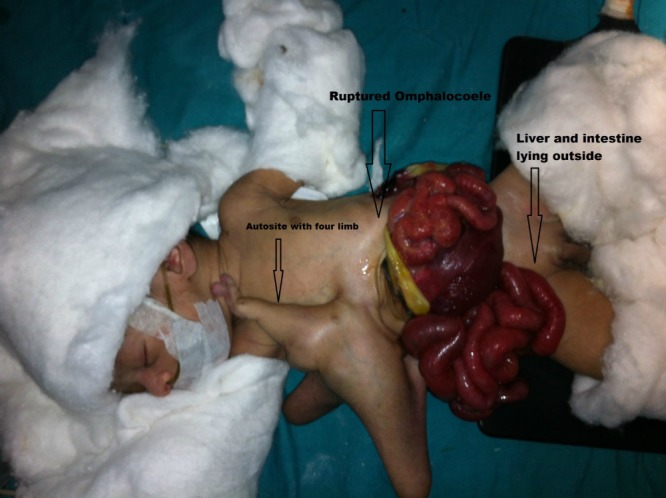
Figure 1: Preoperative image

**Figure:**
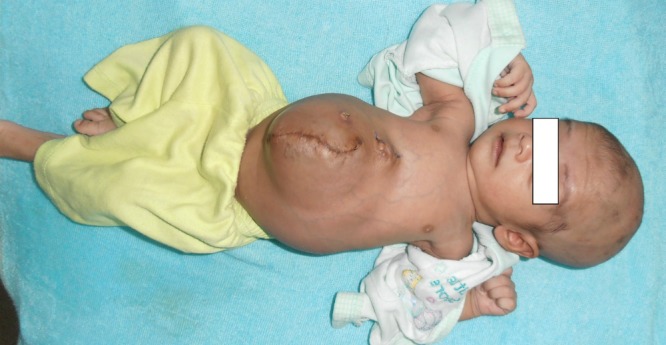
Figure 2: At follow-up

## DISCUSSION

Conjoint twins are a special area of pediatric surgery that require a lot of preoperative work-up to clearly identify the altered anatomy, level of cleavage, associated anomalies, probability of survival of either twins, and postoperative long term outcome. General condition of the patient and associated morbidities might not give enough time to the surgeon for investigations like CT scan, MRI and angiography especially in case where the other twin is malformed and going to be sacrificed and presented with some acute emergency as in our case where there was associated ruptured omphalocele. We have ruled out cardiac anomalies and renal anomalies in our patient with echocardiography and ultrasonography respectively. Ruptured ompahlocoele is a very rare association with parasitic twins and it is a major indication for emergency surgery. About 200 cases of heteropagus twins have been reported in the literature [3,4] including about 46 cases of epigastric Heteropagus.[1-7] However those with ruptured omphalocele are extremely rare.[7]

## Footnotes

**Source of Support:** Nil

**Conflict of Interest:** None

